# Supplementary material for: MALDI-TOF Mass Spectrometry Online Identification of Trichophyton indotineae Using the MSI-2 Application
Source: J Fungi (Basel). 2022 Oct 19;8(10):1103. doi: 10.3390/jof8101103 (PMC9604624; doi:10.3390/jof8101103)
Supplement: Supplementary file 1 [file jof-08-01103-s001.zip › jof-1954252-supplementary.pdf]

Supplemental Table S1: List of references belonging to the *T. mentagrophytes, T. interdigitale*, and *T. indotineae* species. A=MSI-2 database by Jabet *et al.*; B=MSI-2 database, improved with 12 strains from this study; NR = not recorded.

Strain name	Genotype	Database	Host	Localization	Strain origin
**IHEM 28392**	*T. indotineae*	A	Human	NR	Germany
**IHEM 28386**	*T. indotineae*	A	Human	NR	Bahrain
**IHEM 28378**	*T. indotineae*	A	Human	Skin: foot sole infection	Germany
**IHEM 1104**	*T. interdigitale* G-I	A	Environment	Floor of a swimming pool	Belgium
**IHEM 1106**	*T. interdigitale* G-I	A	Environment	Floor of a swimming pool	Belgium
**IHEM 10342**	*T. interdigitale* G-II	A	Human	Skin: foot sole infection	NR
**IHEM 2497**	*T. interdigitale* G-II	A	Human	Skin: foot sole infection	Belgium
**IHEM 2643**	*T. interdigitale* G-II	A	Human	Skin: foot sole infection	Belgium
**IHEM 2771**	*T. interdigitale* G-II	A	Human	Skin: foot sole infection	Belgium
**IHEM 620**	*T. interdigitale* G-II	A	Human	Skin: foot sole infection	Belgium
**IDFP 1**	*T. interdigitale* G-II	A	Human	Skin: arm infection	France
**IDFP 11**	*T. interdigitale* G-II	A	Human	NR	France
**IDFP 16**	*T. interdigitale* G-II	A	Human	Toenail infection	France
**IDFP 2**	*T. interdigitale* G-II	A	Human	Toenail infection	France
**IHEM 1120**	*T. interdigitale* G-II	A	Environment	Floor of a swimming pool	Belgium
**IHEM 584**	*T. interdigitale* G-II	A	Human	Skin: foot sole infection	Belgium
**ROUT 1**	*T. interdigitale* G-II	A	Human	Toenail infection	France
**ROUT 2**	*T. interdigitale* G-II	A	Human	Toenail infection	France
**ROUT 3**	*T. interdigitale* G-II	A	Human	Toenail infection	France
**ROUT 5**	*T. interdigitale* G-II	A	Human	Skin: foot sole infection	France
**ROUT 6**	*T. interdigitale* G-II	A	Human	Skin: foot sole infection	France
**LMA951702**	*T. interdigitale* undetermined	A	NR	NR	NR
**IHEM 3997**	*T. mentagrophytes* G-II*	A	NR	NR	NR
**ENVA-W985**	*T. mentagrophytes* G-II*	A	Dog: Braque	Skin infection	France
**ROUT 13**	*T. mentagrophytes* G-II*	A	Human	ND	France
**ENVA-W1330**	*T. mentagrophytes* G-III	A	Dog: Border Collie	Skin infection	France
**IHEM 14088**	*T. mentagrophytes* G-III*	A	Human	Skin: leg infection	Belgium
**IHEM 4268**	*T. mentagrophytes* G-III*	A	Human	Skin: face infection	Belgium
**IHEM 4270**	*T. mentagrophytes* G-III*	A	Human	Skin: face infection	Belgium
**ENVA-U9307**	*T. mentagrophytes* G-III*	A	Cat	Skin infection	France
**ENVA-W1161**	*T. mentagrophytes* G-III*	A	Dog: Jack Russell	Skin infection	France
**ENVA-W1196**	*T. mentagrophytes* G-III*	A	Cat	Skin infection	France
**ENVA-W1218**	*T. mentagrophytes* G-III*	A	Dog: Jack Russell	Skin infection	France
**ENVA-W1219**	*T. mentagrophytes* G-III*	A	Dog: Whippet	Skin infection	France
**ENVA-W1222**	*T. mentagrophytes* G-III*	A	Dog: Boxer	Skin infection	France
**SAT 7**	*T. mentagrophytes* G-III*	A	Human	Hair	France
**IHEM 19643**	*T. mentagrophytes* G-IV	A	Human	Skin: foot sole infection	France
**IHEM 14015**	*T. mentagrophytes* G-V	A	Sheep	Hair	South Africa
**IHEM 10162**	*T. mentagrophytes* G-XXIV	A	Chinchilla	Hair	Belgium
**IHEM 3289**	*T. mentagrophytes* G-XXIV	A	Human	NR	Belgium
**IHEM 3299**	*T. mentagrophytes* G-XXIV	A	Human	NR	Belgium
**ENVA W1207**	*T. mentagrophytes* undetermined	A	NR	NR	France
**MRS1218158**	*T. mentagrophytes* undetermined	A	Human	NR	France
**RES-AVC92**	*T. indotineae*	B	Human	Skin: inguinal fold infection	France
**SLS-CRTIM10**	*T. indotineae*	B	Human	Skin: inguinal fold / leg infection	France
**PSL5.768**	*T. indotineae*	B	Human	Skin: abdomen infection	France
**RES-AVC30**	*T. interdigitale* G-I	B	Human	Toenail infection	France
**RES-BCL12**	*T. interdigitale* G-I	B	Human	Skin: between toes infection	France
**SLS-CTRL1**	*T. interdigitale* G-II	B	Human	Toenail infection	France
**PSL3.420**	*T. interdigitale* G-II	B	Human	Toenail infection	France
**PSL4.463**	*T. interdigitale* G-II	B	Human	Toenail infection	France
**RES-BCL-173**	*T. mentagrophytes* G-II*	B	Human	Skin: hand infection	France
**RES-HMD-126**	*T. mentagrophytes* G-III*	B	Human	Skin: arm infection	France
**SLS-CTRL7**	*T. mentagrophytes* G-VI	B	Human	Toenail infection	France
**PSL3.316**	*T. mentagrophytes* G-VII	B	Human	Skin: arm infection	France

Supplemental Table S2: List and origin of the 67 isolates constituting assessment panel 1. G = genotype; ND = not documented.

PANEL 1 isolate name	G	host	origin	patient origin / known travel
**IHEM 1106**	I	Environment	Floor of a swimming pool in Belgium	Belgium
**RES-AVC30**	I	Human	Toenail infection	ND
**RES-AVC56**	I	Human	Skin: between toes infection	ND
**RES-BCL12**	I	Human	Skin: between toes infection	ND
**RES-BCL141**	I	Human	Toenail infection	ND
**RES-BCL35**	I	Human	Toenail infection	ND
**RES-SAT28**	I	Human	Skin: foot sole infection	ND
**RES-SAT73**	I	Human	Toenail infection	ND
**IDFP 1**	II	Human	Skin: arm infection	ND
**IDFP 11**	II	Human	ND	ND
**IDFP 16**	II	Human	Toenail infection	ND
**IDFP 2**	II	Human	Toenail infection	ND
**IHEM 1120**	II	Environment	Floor of a swimming pool in Belgium	Belgium
**IHEM 584**	II	Human	Skin: foot sole infection	Belgium
**PSL3.420**	II	Human	Toenail infection	ND
**PSL3.485**	II	Human	Toenail infection	ND
**PSL4.206**	II	Human	Toenail infection	ND
**PSL4.207**	II	Human	Skin: between toes infection	ND
**PSL4.37**	II	Human	Skin: foot sole infection	ND
**PSL4.462**	II	Human	Toenail infection	ND
**PSL4.463**	II	Human	Toenail infection	ND
**PSL6.40**	II	Human	Toenail infection	ND
**PSL6.421**	II	Human	Toenail infection	ND
**ROUT 1**	II	Human	Toenail infection	ND
**ROUT 2**	II	Human	Toenail infection	ND
**ROUT 3**	II	Human	Toenail infection	ND
**ROUT 5**	II	Human	Skin: foot sole infection	ND
**ROUT 6**	II	Human	Skin: foot sole infection	ND
**SLS-CTRL1**	II	Human	Toenail infection	ND
**SLS-CTRL2**	II	Human	Skin: palm/foot sole infection	ND
**SLS-CTRL4**	II	Human	Toenail/foot sole infection	ND
**SLS-CTRL5**	II	Human	Skin: face/between toes infection	ND
**SLS-CTRL6**	II	Human	Toenail/between toes infection	ND
**SLS-CTRL8**	II	Human	Skin: leg infection	ND
**ENVA-W985**	II*	Dog: Braque	Skin infection	ND
**RES-BCL-173**	II*	Human	Skin: hand infection	ND
**ROUT 13**	II*	Human	ND	ND
**ENVA-W1330**	III	Dog: Border Collie	Skin infection	ND
**ENVA-U9307**	III*	Cat	Skin infection	ND
**ENVA-W1161**	III*	Dog: Jack Russell	Skin infection	ND
**ENVA-W1196**	III*	Cat	Skin infection	ND
**ENVA-W1218**	III*	Dog: Jack Russell	Skin infection	ND
**ENVA-W1219**	III*	Dog: Whippet	Skin infection	ND
**ENVA-W1222**	III*	Dog: Boxer	Skin infection	ND
**RES-HMD-126**	III*	Human	Skin: arm infection	ND
**SAT 7**	III*	Human	Hair	ND
**SLS-CTRL7**	VI	Human	Toenail infection	ND
**PSL3.316**	VII	Human	Skin: arm infection	ND
**AVC4**	VIII	Human	Skin: inguinal fold/abdomen/leg infection	Bangladesh
**AVC4b**	VIII	Human	Skin: inguinal fold/abdomen/leg infection	Bangladesh
**AVC6**	VIII	Human	Skin: inguinal fold/abdomen/leg infection	Myanmar
**HOUS3**	VIII	Human	ND	ND
**PSL5.768**	VIII	Human	Skin: abdomen infection	Tibet
**PSL5.769**	VIII	Human	Skin: inguinal fold infection	Tibet
**RES-AVC92**	VIII	Human	Skin: inguinal fold infection	India
**RES-BCH16**	VIII	Human	Fingernail infection	ND
**RES-BCL120**	VIII	Human	Toenail infection	ND
**RES-BCL75**	VIII	Human	Axillary fold infection	ND
**SAT1**	VIII	Human	Skin: inguinal fold/abdomen/leg infection	Bangladesh
**SAT-HOU1**	VIII	Human	Skin: inguinal fold/abdomen/leg infection	Bangladesh

**SEB1**	VIII	Human	Skin: inguinal fold/abdomen / leg infection	Bangladesh
**SLS-CRTIM10**	VIII	Human	Skin: inguinal fold/leg infection	India
**SLS-CRTIM11**	VIII	Human	Skin: thigh/abdomen/arms infection	India
**SLS-CRTIM13**	VIII	Human	Skin: thigh/back/inguinal fold	Tibet
**SLS-CRTIM3**	VIII	Human	Skin: inguinal fold/abdomen/leg infection	India
**SLS-CRTIM4**	VIII	Human	Skin: legs/trunk/arms infection	Bangladesh
**SLS-CRTIM9**	VIII	Human	Skin: legs/arms infection	India

Supplemental Table S3: List of the DNA references used for *Trichophyton mentagrophytes* species complex genotyping.

Accession number	Genotype
FM986691	*Trichophyton interdigitale* genotype I
JX122216	*Trichophyton interdigitale* genotype II
KP132819	*Trichophyton mentagrophytes* genotype II*
FM986750	*Trichophyton mentagrophytes* genotype III
MF926358	*Trichophyton mentagrophytes* genotype III*
KJ606102	*Trichophyton mentagrophytes* genotype IV
KU496915	*Trichophyton mentagrophytes* genotype V
KT285210	*Trichophyton mentagrophytes* genotype VI
KT253558	*Trichophyton mentagrophytes* genotype VII
KT192500	*Trichophyton indotineae*
MK447613	*Trichophyton mentagrophytes* genotype IX
MK312735	*Trichophyton interdigitale* genotype X
MK312755	*Trichophyton interdigitale* genotype XI
MF109039	*Trichophyton interdigitale* genotype XII
MK312917	*Trichophyton mentagrophytes* genotype XIII
MK312950	*Trichophyton mentagrophytes* genotype XIV
MK312937	*Trichophyton mentagrophytes* genotype XV
MK312933	*Trichophyton mentagrophytes* genotype XVI
MK312990	*Trichophyton mentagrophytes* genotype XVII
MK313028	*Trichophyton mentagrophytes* genotype XVIII
MK312878	*Trichophyton mentagrophytes* genotype XIX
MK313030	*Trichophyton mentagrophytes* genotype XX
MK312891	*Trichophyton mentagrophytes* genotype XXI
MK312888	*Trichophyton mentagrophytes* genotype XXII
MK313044	*Trichophyton mentagrophytes* genotype XXIII
AF170453	*Trichophyton mentagrophytes* genotype XXIV
MT858874	*Trichophyton interdigitale* genotype XXV
MT858956	*Trichophyton mentagrophytes* genotype XXVI
